# Cleanroom Maintenance Significantly Reduces Abundance but Not Diversity of Indoor Microbiomes

**DOI:** 10.1371/journal.pone.0134848

**Published:** 2015-08-14

**Authors:** Alexander Mahnert, Parag Vaishampayan, Alexander J. Probst, Anna Auerbach, Christine Moissl-Eichinger, Kasthuri Venkateswaran, Gabriele Berg

**Affiliations:** 1 Institute of Environmental Biotechnology, Graz University of Technology, Graz, Austria; 2 Biotechnology and Planetary Protection Group, Jet Propulsion Laboratory, Pasadena, California, United States of America; 3 Department of Earth and Planetary Sciences, University of California, Berkeley, California, United States of America; 4 Institute for Microbiology and Archaea Center, University of Regensburg, Regensburg, Germany; 5 Medical University Graz, Department of Internal Medicine, Graz, Austria; 6 BioTechMed Graz, Graz, Austria; University of Aveiro, PORTUGAL

## Abstract

Cleanrooms have been considered microbially-reduced environments and are used to protect human health and industrial product assembly. However, recent analyses have deciphered a rather broad diversity of microbes in cleanrooms, whose origin as well as physiological status has not been fully understood. Here, we examined the input of intact microbial cells from a surrounding built environment into a spacecraft assembly cleanroom by applying a molecular viability assay based on propidium monoazide (PMA). The controlled cleanroom (CCR) was characterized by ~6.2*10^3^ 16S rRNA gene copies of intact bacterial cells per m^2^ floor surface, which only represented 1% of the total community that could be captured via molecular assays without viability marker. This was in contrast to the uncontrolled adjoining facility (UAF) that had 12 times more living bacteria. Regarding diversity measures retrieved from 16S rRNA Illumina-tag analyzes, we observed, however, only a minor drop in the cleanroom facility allowing the conclusion that the number but not the diversity of microbes is strongly affected by cleaning procedures. Network analyses allowed tracking a substantial input of living microbes to the cleanroom and a potential enrichment of survival specialists like bacterial spore formers and archaeal halophiles and mesophiles. Moreover, the cleanroom harbored a unique community including 11 exclusive genera, e.g., *Haloferax* and *Sporosarcina*, which are herein suggested as indicators of cleanroom environments. In sum, our findings provide evidence that archaea are alive in cleanrooms and that cleaning efforts and cleanroom maintenance substantially decrease the number but not the diversity of indoor microbiomes.

## Introduction

In recent years much attention has been given to the investigation of the microbiome of the built environment [1]. These are interesting habitats associated with human health [2–4] since humans spend most of their time indoors. In addition to its human residents [5,6] the microbiome of a built environment is determined by numerous environmental parameters: location, usage, architectural design, ventilation and occupancy, as important microbial dispersal vectors [1,7–11]. All studies show that humans are constantly exposed to microorganisms that might affect their microbiome and could potentially have a strong influence on health [12]. Although excessive removal and eradication of mainly beneficial microbes from the indoor environment could have adverse effects [13], some built environments like intensive care units, operating theaters, and particularly cleanrooms need to maintain indoor environments with very low microbial abundance to protect human health or to safeguard the quality of industrial product assembly. An enhanced understanding of the cleanroom microbiome can reveal the effect of microbial control, maintenance, and cleaning on microbial community structure in the built environment.

Cleanrooms represent highly specific environments because many of the environmental parameters are controlled e.g. the amount of particles (via cleaning, disinfection, surface sterilization), type and quality of gaseous substances (air quality is adjusted by filters and adsorption), temperature and humidity (using air conditioning systems), the light source, electrostatics and electromagnetics (can be controlled by deduction, ground connection, ionization and architectural arrangements) (for ISO certified cleanrooms see Online Browsing Platform on ISO standards; https://www.iso.org/obp/ui/#iso:std:iso:14644:-1:ed-1:v1:en). Cleanrooms used for spacecraft assembly have been monitored with special adapted sampling protocols for low-biomass environments in respect to microbial abundance and diversity for many years, as some space missions are subject to planetary protection policy [14–16]. However, complete sterility or absence of biological contamination in spacecraft assembly cleanrooms cannot be achieved during assembly, testing, and launching operations, since some resistant, well-adapted microorganisms are capable of surviving and withstanding harsh cleanroom conditions e.g. continuous HEPA air filtration and very low abundance of particles, water and nutrients [17]. Although humans were identified as the major vector for contamination in cleanrooms [18,19], trained engineers still have to interact closely with spacecraft components to guarantee assembly quality. Since Viking mission days, these cleanrooms have been studied and documented by traditional microbiological methods [20] and later cultivation independent molecular methods were employed as well to understand microbial community structure of the spacecraft and associated surfaces [21,22]. More recently, DNA microarray (PhyloChip) and high throughput sequencing technologies (pyrosequencing) were performed [23–26]; and showed that >90% of the sequences arose from dead and non-viable microbial cells by the application of propidium monoazide (PMA) treatment [25]. PMA is able to mask DNA from dead microbes with permeable cell structures and free extracellular DNA after light activation, for downstream PCR based analysis. Hence, the source of DNA can be determined for all and only intact cells [27].

Consequently, this study has been focused on the potential viable microbiome and its origin by a combination of the DNA masking agent PMA assay [27,28] with Illumina 16S rRNA gene amplicon deep sequencing and qPCR analysis. In addition, an adenosine tri-phosphate (ATP) assay that differentiates between dead organisms and measures only intact and metabolically active cells was used [29] and compared with the PMA-qPCR analysis. Since the changing room could be identified as the main source for microbial contaminants into the cleanroom environment in a prior study [26], we focused on the comparison of microbial abundance and diversity of intact cells between a controlled cleanroom (CCR) and its surrounding uncontrolled adjoining facility (UAF). As a further step, deeper sequencing was applied to reveal the composition of underrepresented microbes and assess their viability status. This deep diversity analysis should add another puzzle to the picture of strict controlled built environments and estimate its risks to jeopardize future life detection missions.

## Materials and Methods

### Environmental conditions

**Fig 1:**
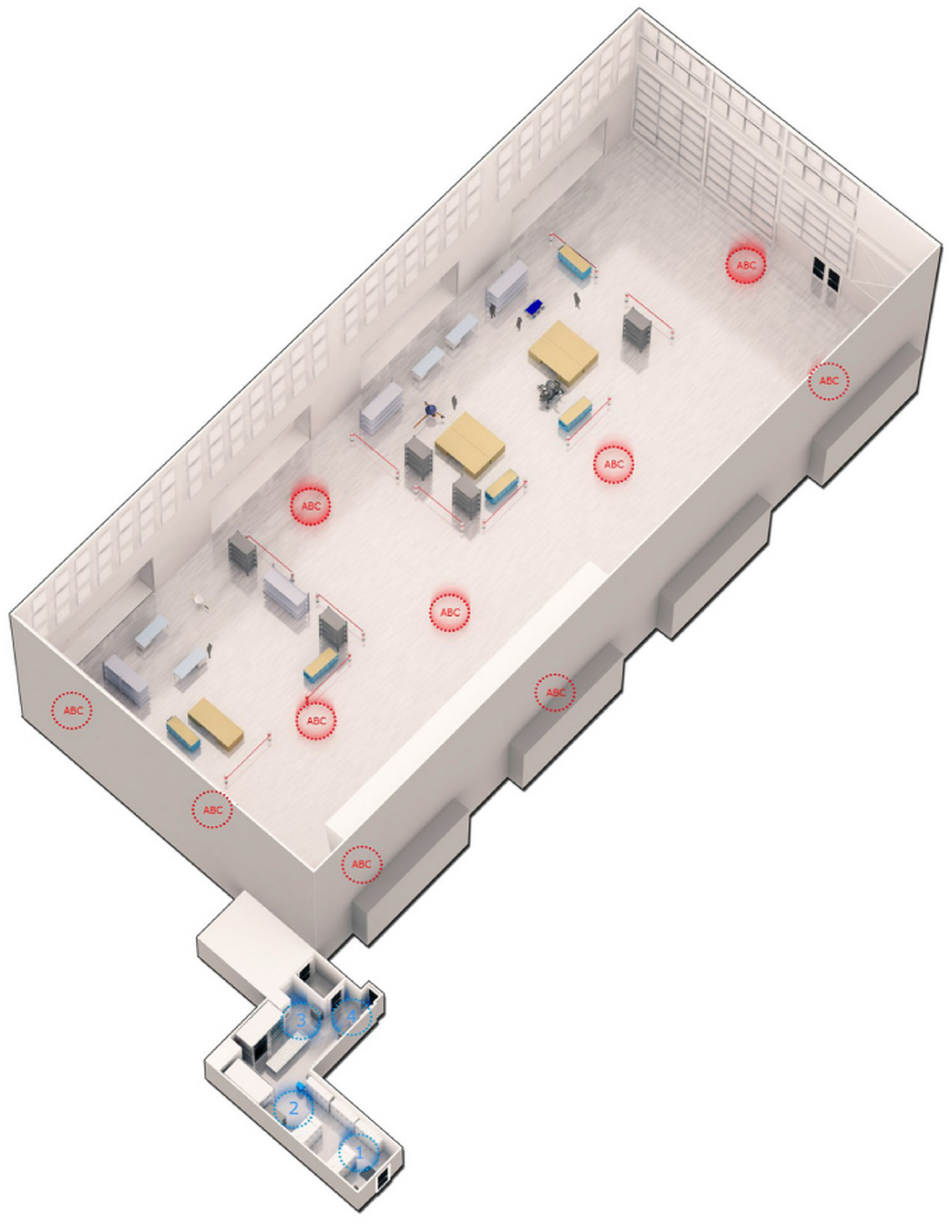
3D-rendered-model. of sampled controlled cleanroom (CCR) and its uncontrolled adjoining facility (UAF). Triplicate of ten different locations in CCR are indicated by A, B and C (red circles). Samples from four individual locations in UAF are numbered 1 to 4 (blue circles).

The relatively small gowning area (45.9 m^2^, Fig 1) was the main entrance for staff to have access into the particulate controlled cleanroom (CCR, spacecraft assembly facility–SAF at NASA Jet Propulsion Laboratory). Sticky mats in front of and behind doors were placed to remove dirt from the soles of shoes. The gowning area was equipped with lockers to keep the street clothes in before the donning of cleanroom garments. Before entering the air shower, personnel were required to clean their footwear by means of a shoe cleaner. Gowning with cleanroom certified textiles (coat, head cover, goggles, surgical mask, gloves, shoe-cover) was strictly followed and then staff members were allowed to pass through air shower systems before entering. Within the cleanroom, staff members were advised to perform their duties with slow body movements to avoid the spreading of human and floor associated particles. The cleanroom (ISO8, 100K, 921.1 m^2^) itself had more than 20 times the floor surface compared to the gowning area. In both rooms, floor surfaces consisted of polymer plastic materials (vinyl composition tiles) and were illuminated by artificial light sources. Access to the cleanroom was strictly controlled, air was continuously exchanged through HEPA filter systems, temperature and humidity were constantly automatically adjusted, numbers of particles with a size ≥0.5 μm were counted, and floors were regularly cleaned up to several times per week. Sampling was performed prior to the regular cleaning schedule. Both rooms were cleaned on September 24^th^ and on October 3^rd^ 2012 using Kleenol 30 (Mission Kleensweep Products Inc., Los Angeles, USA). Kleenol 30 is a highly alkaline (11.5 to 12 pH) product, which consists of (v/v) 1% dodecyl benzene sulfonic acid, 1 to 4% silicic acid disodium salt, 12.5% ethanol, 1 to 5% nonylphenol ethoxylate, a non-ionic surfactant, dissolved in distilled water. During the period of sampling time the cleanroom did not harbor mission critical spacecraft hardware. The sampling team included four persons (three scientists and one person from the cleanroom managing staff). At the time of sampling, in addition to the sampling team, two assembly technicians were present in the CCR, whereas in the UAF only sampling team members were present. Particles smaller than 0.5 μm in size were far below the required specification for this CCR cleanroom (100K) during sampling operations (max. 570 particles per cubic foot; 20,129 particles per m^3^).

### Sampling procedure

While sampling, the ATP content was measured with a hand-held ATP device in real-time as described elsewhere [29] to identify hot-spots of microbial contamination and reveal the gross contamination of the surface to be sampled (Table A in S1 File). Subsequently, various cleanroom floor locations of the CCR and UAF were sampled. One square meter surface area of three adjacent places (A, B, C; Fig 1) of 10 different CCR locations was sampled. Each adjacent area of all 10 locations was pooled (3 replicates each containing 10 m^2^) and analyzed for molecular analyses. Compared to UAF, ~10 times more surface area was collected from CCR, since it was reported that microbial abundance was ~10-fold less in a Class 100K (ISO 8) cleanroom compared to uncertified ordinary rooms [24]. Hence, when samples were taken from UAF only one square meter surface area of 4 different locations was sampled (Fig 1). Field blanks were collected without touching actual floor surfaces to reveal the microbial background contaminants from other sources than the floor surfaces (e.g. from the indoor air).

Surface sampling of the floor (1 m^2^) was performed with adapted protocols for low-biomass environments using pre-moistened biological sampling kits (BiSKits; QuickSilver Analytics, Abingdon, MD) as described previously [30]. Prior to sampling, all the BiSKit devices were rinsed with 15 mL of DNA-free phosphate buffered saline (PBS), which served as controls throughout the experiments. The manufacturer-provided buffer was discarded and sterile PBS was used and then added to the macrofoam sponge component of the BiSKit. Once the PBS had adequately absorbed to the macrofoam sponge, the sampler was unscrewed from the module and was traversed about the surface area of interest (ca. 1 m^2^), first in a horizontal fashion, then vertically, and finally in a diagonal sweeping pattern. Immediately following the collection of a sample from the surface, the macrofoam sponge sampler was forcefully screwed back into the BiSKit module so as to squeeze as much sample as possible from the sponge into the collection tube. The module and attached collection tube were then transported in a sealed bag to the laboratory, where they were further processed under a biohood. Upon return to the lab, the PBS was recovered from the BiSKit sponge by screwing the sponge casing against the BiSKit cover several times, allowing samples to be collected into the attached sample bottle.

### Concentration of biomolecules

The pooled fractions of the samples were aseptically transferred to Amicon Ultra-15 centrifugal filter tubes (Ultracel-50 membrane, catalog no. UFC905096; Millipore, Jaffrey, NH), which were in turn placed within a Sorvall RC-5B refrigerated centrifuge (Thermo Scientific, Waltham, MA) and spun at 4,000 g for 10 min. Each filter unit has a molecular mass cutoff of 50 kDa, which facilitates the concentration of bacterial cells, spores, and exogenous nucleic acid fragments greater than 100 bp into a final volume of ca. 1.5 ml. The resulting volume was aseptically transferred to a sterile microcentrifuge tube. A comparable amount of sterile PBS was concentrated in a separate filter tube, serving as a negative control for each concentration/extraction.

### Sample processing

All filtered samples were then divided into three separate aliquots: one of the aliquots (500 μl) was subjected to PMA pretreatment (viability assessment), the second (500 μl) was an untreated environmental sample (viable + nonviable cells; total DNA), and the third (500 μl) was used for ATP analysis (see below). One 500 μl aliquot of filter-concentrated sample suspension was treated with PMA (Biotium, Inc., Hayward, CA, USA) to a final concentration of 50 μM [27,31], followed by thorough mixing and incubation in the dark for 5 min at room temperature. The sample was exposed to PhAST blue-Photo activation system for tubes (GenIUL, S.L., Terrassa, Spain) for 15 min (in parallel with the non-PMA treated sample). Samples were then split in half and one half was subjected to bead beating with the Fastprep-24 bead-beating instrument (MP Biomedicals, Santa Ana, CA, USA) with parameters set at 5 m/s for 60 s. The second half of the unprocessed sample was then combined with the mechanically disrupted counterpart to allow microbial yields from hardy cells and spores with a limited loss of the overall microbial diversity. DNA was extracted via the Maxwell 16 automated system (Promega, Madison, WI, USA), in accordance with manufacturer’s instructions, and resulting DNA suspensions (100 μl each) were stored at −20°C.

### ATP-assay

A bioluminescence assay was performed to determine the total ATP and intracellular ATP from all samples using the CheckLite HS kit (Kikkoman), as described previously [29]. Briefly, to determine total ATP (dead and viable microbes), sample aliquots were combined with an equal volume of a cell lysing detergent (benzalkonium chloride) and incubated at room temperature for 1 min prior to the addition of a luciferin–luciferase reagent. The sample was mixed, and the resulting bioluminescence was measured with a luminometer (Kikkoman). For intracellular ATP measures of intact microbes a tenth volume of an ATP-eliminating reagent (apyrase, adenosine deaminase) was added to the sample and allowed to incubate for 30 min to remove any extracellular ATP. After extracellular ATP removal the assay for ATP was carried out, as described above, including sterile PBS as a negative control. As previously established, 1 Relative Light Unit (RLU), the unit of ATP measurement, was assumed to be approximately equal to 1 CFU [17].

### Quantitative PCR

Quantitative PCR was accomplished as described [32]. One μl of extracted DNA (< 1 ng—> 10 ng) was used for a 20 μl reaction mix containing 10 μl SYBR Green Supermix (BIO-RAD, Hercules, CA, USA), 0.5 μl forward and reverse primers (1369F and 1492R; [33]) at a concentration of 18 μM and 8 μl of PCR grade water. DNA templates were amplified with 16S rRNA gene standards from *Bacillus subtilis* using a BIO-RAD C1000 qPCR thermal cycler in three replications and the following program: 95°C for 3 min, 95°C for 15 sec., combined annealing and extension at 55°C for 35 s, steps repeated for 39 times and finished with a step at 95°C for 10 sec, and a final elongation from 65°C– 95°C by an increase of 0.5°C for 5 sec. respectively.

16S rRNA gene amplicon Illumina MiSeq deep sequencing: For 16S rRNA gene PCR, amplicons of samples and controls were generated with universal primers 515F and 806R [34–36] using 7–10 bp barcodes for identification in paired-end Illumina MiSeq runs [37,38]. One μl extracted DNA was used as a template in a 10 μl PCR reaction containing 5.5 μl PCR grade water, 2 μl 5xPhusion HF buffer, 1 μl 2 mM dNTPs, 0.2 μl of primers 515F and 806R (0.2 μM final conc.) respectively and 0.1 μl Phusion Polymerase. PCR settings were as follows: 98°C 30 s, 35 cycles of 98°C 10 sec, 50°C 30 sec, 72°C 10 sec and a final extension at 72°C for 10 min. Illumina barcodes were attached to PCR products in a subsequent 20 μl PCR reaction with 20 cycles of 98°C 10 sec, 60°C 30 sec, 72°C 11 sec. Barcoded products of four individual PCR reactions were pooled, cleaned with Wizard SV Gel and PCR Clean-Up System kit (Promega, Madison, WI, USA) and checked finally via gel electrophoresis. Resulting DNA concentrations were determined on the NanoDrop instrument (Thermo Scientific, Wilmington, DE, USA). Equimolar concentrations of amplicons were sent to LGC Genomics GmbH, Berlin, Germany for 2x 250 bp paired-end Illumina MiSeq deep sequencing using V2 chemistry. Sequences are deposited in the European Nucleotide Archive (www.ebi.ac.uk) under project PRJEB8763 (ERP009799).

### Bioinformatics

Sequencing reads were demultiplexed with Illumina CASAVA analysis software. Adapters were clipped and reads with < 20 bp were removed. Corresponding forward and reverse reads were stitched into longer fragments using FLASH (overlap 10 bp, max. mismatch 0.25). Amplicons of samples and controls were further sorted by removing reads without barcodes, single reads (only one barcode) and barcode chimeras (different barcodes on 5 and 3 prime site). Resulting reads were quality filtered for deep diversity analysis with QIIME [39] at phred score q30 [40], 5’-3’ orientated, labeled and additional quality filtered using default settings in QIIME. Reads in respective negative controls of each BiSKit sample were removed by a 100% aligned BLAST hit prior to OTU picking (see Table B in S1 File for details on all reads). OTUs were checked for chimeric sequences via ChimeraSlayer, clustered at 97% similarity level [41], taxonomy was assigned with the RDP classifier [42] and a phylogenetic tree was calculated [43]. The resulting rarefied OTU table served as a basis for following alpha and beta diversity analysis and comparative statistics. Possible functions of this marker gene analysis were predicted with PICRUSt [44] according to the tutorial (http://picrust.github.io/picrust/index.html) and Galaxy modules provided by the Huttenhower lab. Core OTUs were calculated in QIIME and served as input for the network analysis. For clustering of OTUs in the network a stochastic spring-embedded algorithm was used to generate node and edge tables and calculate eweights for each OTU. The bipartite core OTU network was then visualized in Cytoscape 2.8.3 [45] with the edge-weighted spring embedded eweight layout and customized node tables for further node size (relative abundance of OTUs), color (sample color and mixtures according to Itten’s color circle) and labeling adjustments (taxonomic assignment on genus level). Finally, edge width and opacity was correlated with respective eweights.

Statistical analysis: Non-metric multidimensional scaling, hierarchal clustering using the average neighbor algorithm, and multi-response-permutation-procedure (MRPP, 999 monte carlo permutations) were conducted in the R programing environment in conjunction with the vegan package [46], as was analysis of variance (ANOVA) for univariate analysis of data [46,47]. Analysis of Similarities (ANOSIM) and two-sided Pearson correlation with Fisher’s z-transformation were conducted in QIIME [39].

## Results and Discussion

The comparison of a controlled cleanroom (CCR) with its uncontrolled adjoining facility (UAF) revealed new insights into the potential viable microbiome exposed to strict environmental control and regular cleaning within the built environment on the level of microbial abundance and diversity.

### Microbial abundance of intact cells is highly reduced in a controlled cleanroom

**Fig 2:**
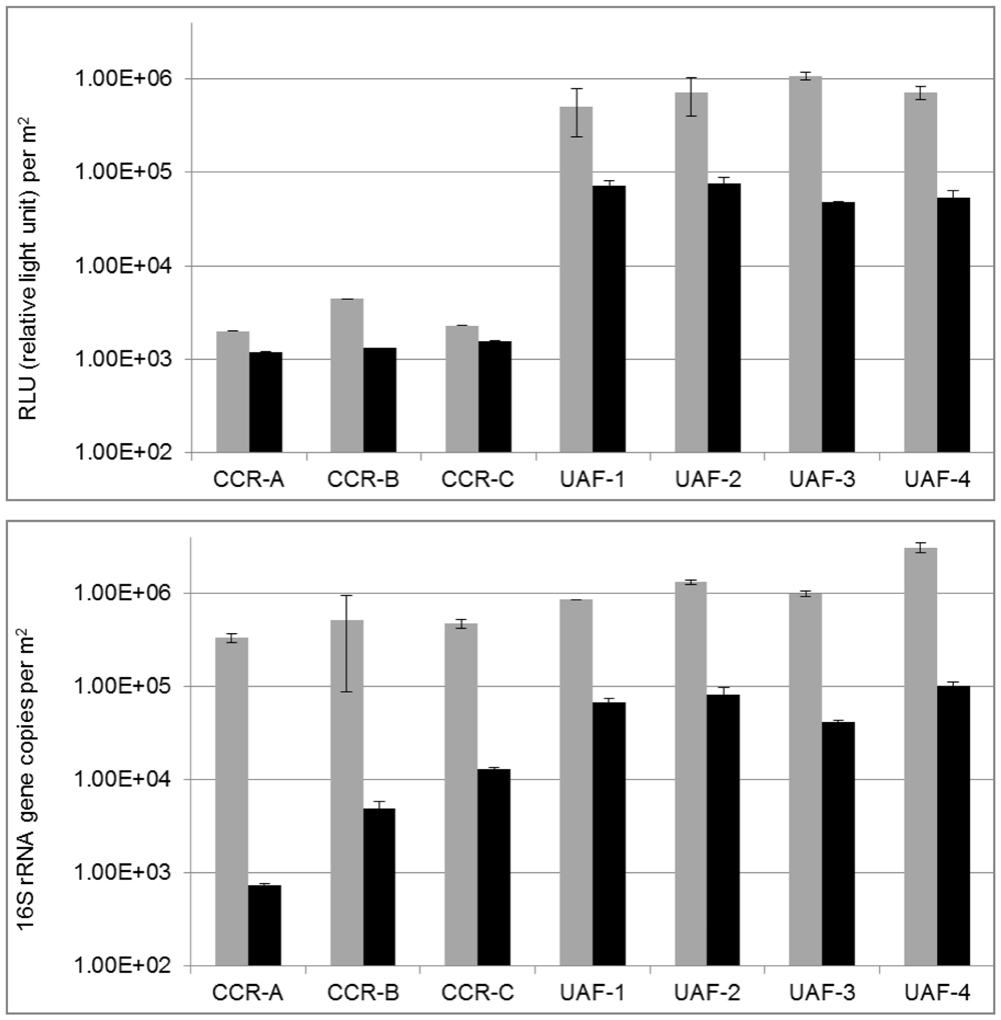
Quantitative evaluations. of controlled cleanroom (CCR) and uncontrolled adjoining facility (UAF) per square meter floor surface. Upper panel shows ATP counts for total (grey bars) and intracellular ATP (black bars) in CCR and UAF (total and intracellular ATP was determined in duplicate). Lower panel shows 16S rRNA gene copies in CCR and UAF with (black bars)–and without (grey bars) PMA (propidium monoazide) treatment in triplicate (error bars represent positive and negative standard deviations).

As indicated by results of qPCR measurements (Fig 2), an excessive number (~ 1 log) of intact microbial cells were removed by intensive cleaning procedures from the CCR (controlled cleanroom, 6.2 * 10^3^ 16S rRNA gene copies per m^2^) compared to the UAF (uncontrolled adjoining facility, 7.3 * 10^4^ 16S rRNA gene copies per m^2^). The small but intact microbial cell fraction was still metabolically active as determined by ATP measurements (see Fig 2; 1.4 * 10^3^ RLU (relative light unit / m^2^ for CCR and 6.2 * 10^4^ RLU / m^2^), although ATP counts of the overall gross contamination (determined with the handheld device to measure ATP content) were much lower in the CCR than in UAF (> 4 fold; Table A in S1 File). Analysis of variances (ANOVA) showed significant differences between sample groups (Table C–H in S1 File) for ATP (P = 4.27*10^−9^), qPCR (P = 1.66*10^−13^), ATP vs. qPCR (P = 2*10^−16^), ATP vs. PMA (P = 0.02), and total (entire ATP and DNA) vs. living (intracellular ATP and DNA from intact cells; P = 1.21*10^−9^) data. On the contrary a different picture was visible without PMA treatment or the removal of extracellular ATP. In this case only minor differences could be determined between CCR and UAF regarding qPCR measurements and the total microbial portion due to many amplified sequences from dead cells (4.4 * 10^5^ for CCR and 1.6 * 10^6^ 16S rRNA gene copies for UAF per m^2^). However, the total ATP content differed by ~2 logs between CCR (2.9 * 10^3^ RLU / m^2^) and UAF (7.5 * 10^5^ RLU/m^2^) samples due to a reduced metabolic activity of dead cells, lower half-life of ATP compared to DNA or the alkaline cleaning reagents (Kleenol 30, pH 12) simply removed ATP but not DNA. The ATP-assay is applicable as a universal marker of metabolic activity for viable cells, but is strongly dependent on the physiological status, size and energy metabolism of microbes, which could bias estimations for microbial abundance per se [29]. Whereas quantitative molecular measures based on nucleic acids have the advantage of targeting microbes beyond the limitations of cultivation, these methods do not differentiate between DNA molecules derived from an extracellular origin or between dead and intact cells, which could potentially lead to overestimation of microbial abundance and diversity [25]. However, PMA helps to distinguish between gene copy counts and reads obtained from dead or intact cells according to the configuration of a microbial cell structure [27,28]. By coupling PMA treatment of samples with qPCR [31], measured 16S rRNA gene copy numbers can be distinguished between all and only intact cells. Although limitations are known for multi–layered cell structures such as microbial spores and in the presence of a high osmolarity [31,48]. Nevertheless, qPCR could be brought into relation with quantitative results of the intracellular ATP content. The equation of intracellular ATP with PMA-qPCR was surprisingly balanced P = 0.02 (total ATP vs. qPCR P = 2*10–16, and total vs. living P = 1.21*10^−9^). The proportion of disintegrated cells or free DNA was higher (99%) inside CCR than in UAF (P = 1.66*10^−13^) and we speculate that the turn-over of extracellular DNA or dead microbes seemed to be much more limited in a cleanroom environment. This might have been due to decreased microbial activity which was also indicated by low levels of ATP counts in the cleanroom. Due to many controlled environmental parameters including temperature, humidity, the tolerated amount of particles, stringent and regular cleaning procedures, as well as restricted access of staff and spacecraft materials, introduced microbes have to face an extreme environment, where most of them have to wait for better conditions referred to by Carrie Arnold as “waiting rooms” for microbes [13].

### Microbial diversity is similar in CCR and UAF

Sequencing of the 16S rRNA gene amplicon pool on Illumina MiSeq resulted in an average yield of 2.98 * 10^4^ reads, whereas controls provided the lowest number of reads per sample and non-PMA treated samples provided the highest number of reads per sample (read statistics are summarized in Table B in S1 File). After quality filtering, chimera removal (~10%) and subtraction of reads matched with controls (13.5%; sequences with a BLAST hit of 100% were removed) [49], a range of 1.0 * 10^4^ up to 5.93 * 10^4^ quality sequences could be retrieved for further analyses.

In general microbial diversity was similar in the CCR and UAF area (average Shannon-Wiener index H’: 6.6 in CCR, 6.8 in UAF), but was reduced by PMA treatment, which resulted in H’: 5.8 for CCR+ and H’: 6.0 for UAF+. This reduction was stable irrespective of the analyzed type of filtered data (Table I in S1 File).

### The microbiome of intact cells in the controlled and uncontrolled built environment shows higher similarity than its total captured microbiome

**Fig 3:**
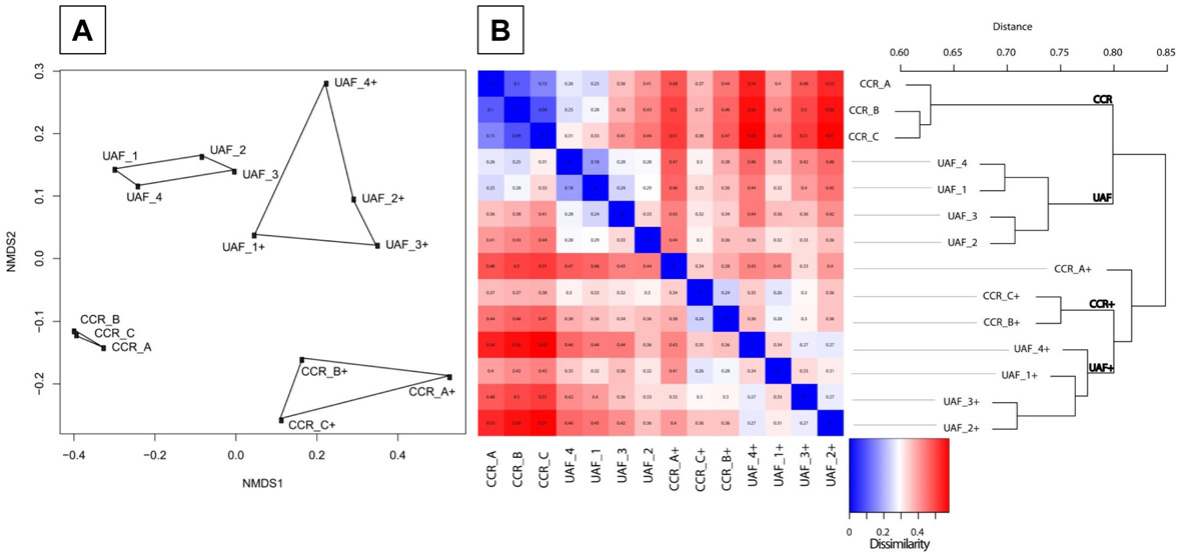
Beta-diversity (unweighted). (A) NMDS plot based on unweighted (left) unifrac distance matrix of rarefied OTUs to 10,011 sequences. Samples treated with PMA prior to DNA extraction are indicated by a plus symbol. CCR: controlled cleanroom. UAF: uncontrolled adjoining facility. Variances are explained per each axis (NMDS1 and NMDS2, Stress = 0.06). (B) Distance based comparison heatmap combined with a hierarchical cluster analysis based on average neighbor (HCAN) of unweighted unifrac distances. Dissimilarity of samples is indicated by a color gradient from blue (similar) via white to red (dissimilar). Samples treated with PMA prior to DNA extraction are indicated by a plus symbol. CCR: controlled cleanroom. UAF: uncontrolled adjoining facility.

Nonmetric multidimensional scaling (NMDS) plots based on unweighted unifrac distance matrices of rarefied OTUs to 10,011 sequences clustered CCR triplicate and samples from UAF separately (Fig 3A). CCR samples showed a higher intragroup similarity compared to UAF samples and samples treated with PMA formed more loose clusters for both rooms. Samples from the entranceway of the uncontrolled UAF facility (UAF_1) and samples from its exit (UAF_4) into the CCR clustered closer than those samples, which were obtained from the center of the room of the uncontrolled UAF area (UAF_2 and UAF_3).

A distance-based comparison heatmap combined with an HCAN cluster dendrogram based on unweighted unifrac distances confirmed results from the NMDS analysis (Fig 3B). Similar clusters formed in both types of analysis when weighted measures were applied (S1A and S1B Fig) and if respective controls where not removed from actual samples (S2 Fig).

The grouping of samples by their respective categories (CCR, CCR+, UAF, UAF+, and controls) was significant, as determined by an ANOSIM-test R = 0.86 (P = 0.001, alpha = 0.05). Monte-carlo permutation-based analysis (MRPP) between CCR samples and UAF samples revealed a delta of 0.022 with a chance corrected within-group agreement of 0.02, indicating minor but significant differences between the sampled community structures. Even higher significant differences between PMA and non-PMA treated community structures were observed with the MRPP (delta = 0.001; chance corrected within-group agreement = 0.05). The phylogenetic composition of samples produced significant correlations using a two-sided Pearson test with Fisher’s z-transformation for CCR_A-B and CCR_B-C, UAF samples 1–4 and when samples with and without their respective control were correlated.

### Many dominant OTUs comprised sequences from dead cells

Microbes facing these harsh conditions were partly unique and highly diverse. In detail, the 16S rRNA gene amplicon pool contained 23 bacterial and 2 archaeal phyla in total. Three OTUs could not be classified to phylum level or a higher resolved order (one not even to a domain of life). These unclassified groups had higher abundances in UAF (1% CCR, 4% UAF) and their relative amount increased in respective samples treated with PMA (2% CCR+, 13% UAF+). Altogether, 337 OTUs could be assigned to genus level (Table J in S1 File). These sequences were dominated by *Actinobacteria* (*Corynebacterium*), *Bacteroidetes* (*Pedobacter*), *Cyanobacteria* (*Chroococcidiopsis*), *Firmicutes* (*Staphylococcus*), *Fusobacteria* (*Leptotrichia*), *Proteobacteria* (*Phenylobacterium*, *Skermanella*, *Sphingobium*, *Shewanella*, *Rickettsiella*, *Halomonas*, *Acinetobacter*, *Pseudomonas*, *Pseudoxanthomonas*), *Verrucomicrobia* (*Prosthecobacter*) and the phylum WPS-2. Twenty of these taxa were only present in CCR e.g. *Prosthecobacter* and WPS-2, and 138 of them were only present in UAF and UAF+ (PMA treated UAF samples) e.g. *Leptotrichia*, *Skermanella*, and *Rickettsiella* (S3 Fig).

Likewise differences in PMA treated (PMA+) and non-treated samples of these dominant sequences could be observed. Similar numbers of genus level assignable OTUs appeared in PMA treated samples (33 CCR+, 38 UAF+), whereas untreated samples harbored ~ 4.6 (CCR) to ~ 6.9 (UAF) times more OTUs resolved to genus level. Overall, PMA treated samples showed sequences belonging to 70 underrepresented taxa (32 genera), which would have been masked by the predominant dead microbial species without any PMA application (Table K in S1 File). The genus *Acinetobacter*, *Pedobacter*, *Phenylobacterium*, *Sphingobium*, *Pseudoxanthomonas*, *Prosthecobacter* and the WPS-2 phylum predominantly comprised sequences from disintegrated cells, whereas sequences from *Corynebacterium*, *Chloroplasts*, *Staphylococcus*, *Skermanella*, *Shewanella*, *Rickettsiella*, *Halomonas* and *Pseudomonas* arose from intact cells (Table J in S1 File).

### The microbiome of intact cells spread to a larger extent between the controlled and uncontrolled built environment

**Fig 4:**
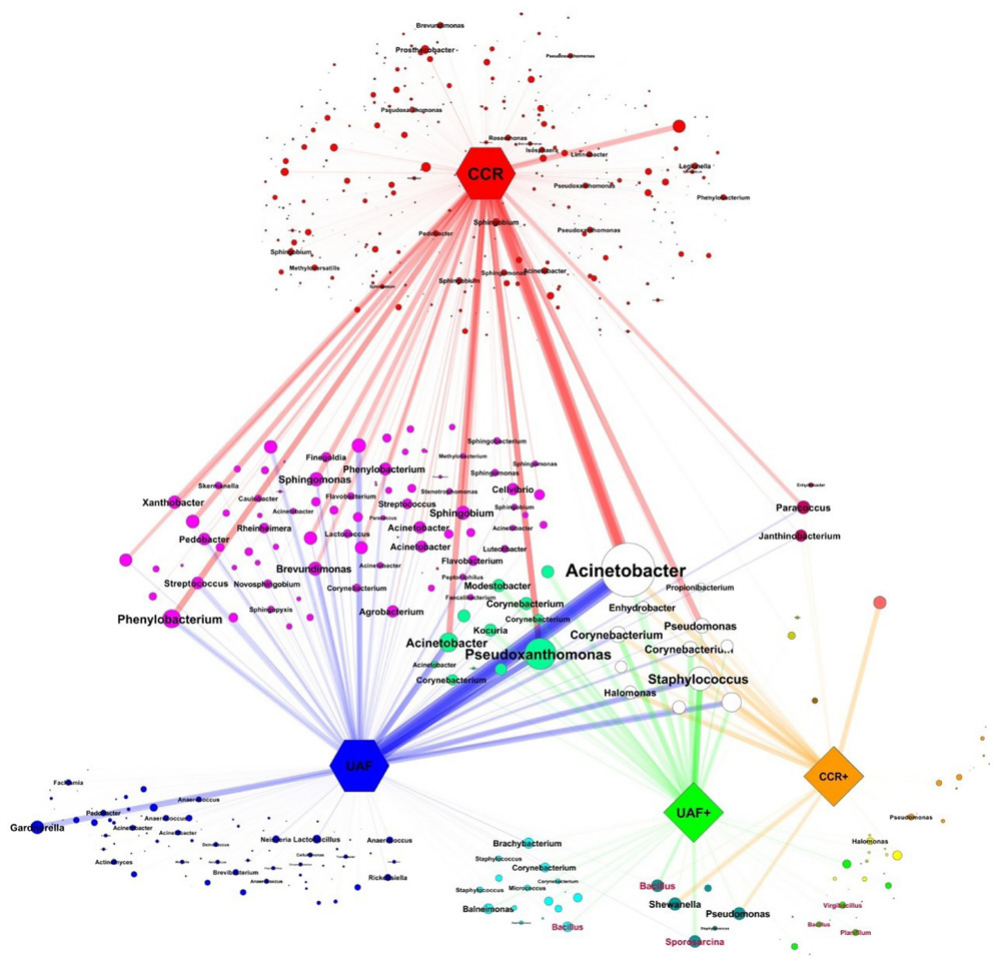
Core OTU network (spring embedded eweighted) of CCR (red), CCR+ (orange), UAF (blue) and UAF+ (green) samples. Node size represents OTU abundance and edge width and opacity is weighted. OTUs resolved to genus level are highlighted and font size correlates with OTU abundance. Bacterial genera in red represent potential spore formers. Samples treated with PMA prior to DNA extraction are indicated by a plus symbol. CCR: controlled cleanroom. UAF: uncontrolled adjoining facility.

Network analysis of the core OTUs (100% present in one sample category irrespective of the other category; see Fig 4) showed different amounts of OTUs shared between CCR replicates, samples from different UAF locations, and their PMA treated fractions (CCR+ and UAF+). Whereas most OTUs of the CCR triplicate were low in abundance (indicated by respective node and font size), those OTUs shared between multiple sample categories revealed higher abundances in 16S rRNA gene amplicons e.g. *Acinetobacter* and *Pseudoxanthomonas*. However, these high abundant OTUs were composed mainly of dead cells. Core OTUs of the CCR shared only a fraction (8.2%) with core OTUs from UAF and revealed clearly which microbes were introduced from outside into the cleanroom environment (49.6%) and that the microbiome of intact cells was spread to a larger extent between both environments (CCR+: 52.4%, UAF+: 69.4%). Samples treated with PMA not only formed their own cluster in the network and ordination analysis, but also comprised most bacterial genera, which are able to form spores (e.g. *Bacillus*, *Sporosarcina*, *Virgibacillus*, *Planifilum*—highlighted in red) beside other genera. The high spreading potential of the intact microbiome may be due to the above-average proportion of survival specialists such as spore forming bacteria, which may abandon their dormant state during more suitable temporal conditions.

### Underrepresented OTUs and taxa could be detected by the application of PMA

**Fig 5:**
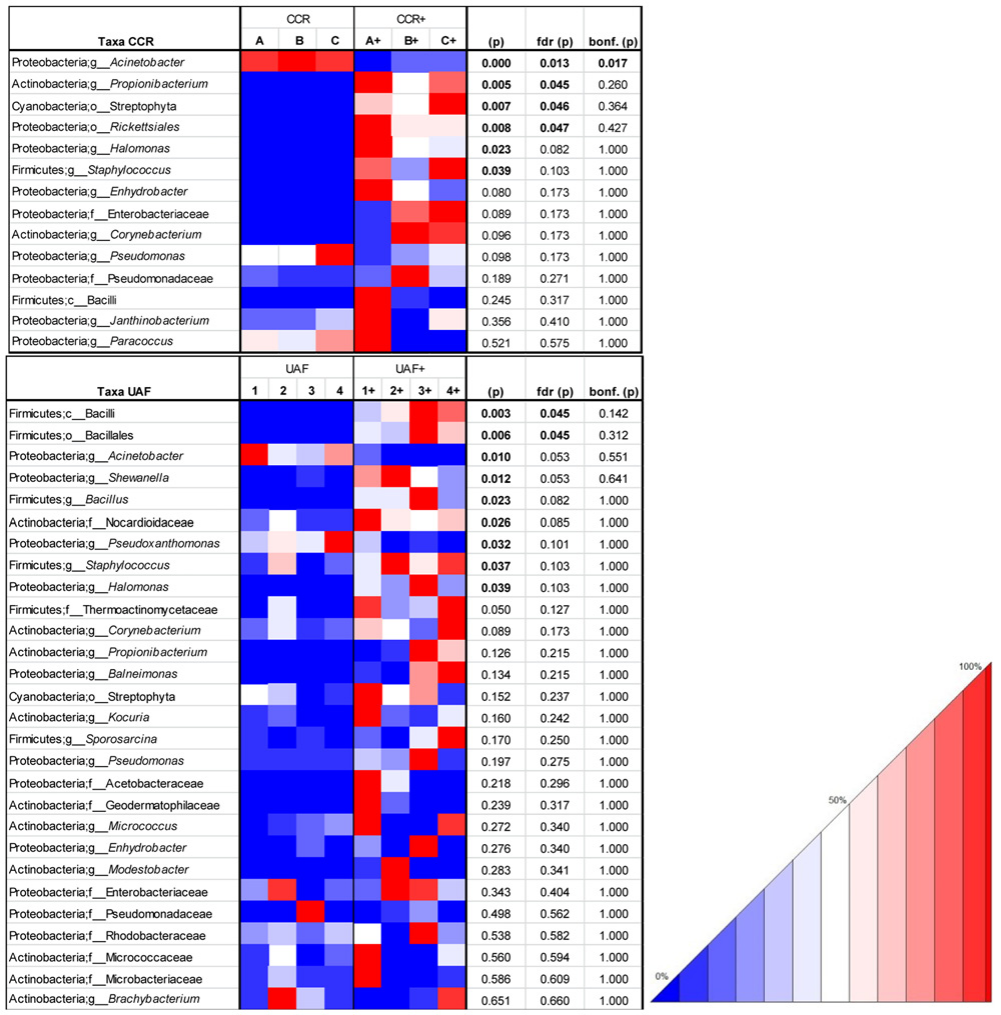
OTU heatmap. based on taxa, which are part of the respective core microbiome from CCR (controlled cleanroom) and UAF (uncontrolled adjoining facility). Color code from blue via white to red (0–50–100%) gives relative amount [%] of respective taxonomic group. Table was rarefied to 3406 OTUs (CCR samples), and 6665 OTUs (UAF samples). Table was sorted according to resulting P-values (p) of an ANOVA test (significant (p) at an alpha of 0.05 are highlighted in bold). (p) were corrected with false discovery rate (fdr (p)) and bonferroni (bonf. (p)). Samples treated with PMA prior to DNA extraction are indicated by a plus symbol.

Some OTUs within the core microbiome showed a relative increase after being treated with PMA (see heatmaps Fig 5, S3 Fig and Table L and M in S1 File). An analysis of variance (ANOVA) showed significant increase of sequences for *Propionibacterium* (P = 0.006) and *Rickettsiales* (P = 0.008) in CCR, bacilli (P = 0.003), Bacillales (P = 0.006), *Shewanella* (P = 0.012), *Bacillus* (P = 0.023) and Nocardioidaceae (P = 0.026) in UAF, *Staphylococcus* (P = 0.039) and *Halomonas* (P = 0.039) after PMA treatment. On the contrary many dominant OTUs e.g. *Acinetobacter* (P = 0.01) and *Pseudoxanthomonas* (P = 0.032) decreased significantly after applying PMA before DNA extraction. Hence, these increasing OTUs seem to prevail as intact cells (or spores) in the core microbiome and those decreasing OTUs showed that a big part of dominant sequences arose from disintegrated cells in both environments. Notably, 11 genera (*Haloferax*, *Microlunatus*, *Virgibacillus*, *Sporosarcina*, *Balneimonas*, *Amaricoccus*, *Aquabacterium*, *Tepidimonas*, *Tolumonas*, *Shewanella*, *Rhodanobacter*) were only present in PMA treated samples from the CCR and could not be detected in the UAF of the spacecraft assembly facility.

Moreover, by its selective DNA masking properties, PMA removed dominant extracellular and DNA from compromised cells to such an extent that underrepresented taxa could be detected in the amplicon pools. The rare biosphere is not only of interest for estimating the biotechnological potential of an ecosystem [50–52], but also it is of great importance for projects related to planetary protection. By providing a highly accurate picture of microbial abundance and diversity in spacecraft assembly cleanrooms, analysis of the rare biosphere would greatly assist in assessing the risk of false positives in future live detection missions in addition to standard bioburden control protocols. Therefore, high quality thresholds for sequence filtering were set as recommended by [40], but single–and doubletons were kept in contrast to other studies, since 85% of these rare OTUs could be classified to genus or family level (Table N in S1 File).

### PMA treatment helps to analyze the rare biosphere

Rarefaction analysis of all samples showed poor coverage (mean 40% ± 9) due to the immense amount of rare abundant OTUs (S4 Fig) as 99% of the microbial diversity was expressed in only 1% of the total abundance. This deep diversity analysis (less than 1% relative abundance) revealed sequences assigned to *Thaumarchaeota* (Candidatus *Nitrososphaera*), *Euryarchaeota* (*Haloferax*), *Armatimonadetes* (*Fimbriimonas*), *Chlamydiae* (Candidatus *Protochlamydia*, Candidatus *Rhabdochlamydia*), *Chlorobi*, *Chloroflexi*, *Fusobacteria* (*Fusobacterium*, *Leptotrichia*, *Sneathia*), GN02, *Gemmatimonadetes*, *Nitrospirae* (*4–29*, *Nitrospira*), *Planctomycetes* (*Gemmata*, *Isosphaera*, *A17*, *Pirellula*, *Planctomyces*), TM6, TM7, *Tenericutes* (*Mesoplasma*, *Mycoplasma*, *Ureaplasma*), *Thermi* (*Deinococcus*, *Thermus*) and *Verrucomicrobia* (*Opitutus*, *Luteolibacter*, *Prosthecobacter*, Candidatus *Xiphinematobacter*, *Ellin506*), WPS-2, and WYO. Part of this deep diversity harbored sequences from intact cells: *Thaumarchaeota* (*Nitrososphaera*), *Euryarchaeota* (*Haloferax*), *Planctomycetes* (*Gemmata*, *Isosphaera*, *A17*), *Thermi* (*Deinococcus*) and *Verrucomicrobia* (*Luteolibacter*, Prosthecobacter).

These rare taxa comprised genera with potential human or plant pathogenic properties like *Afipia*, *Cupriavidus*, *Erwinia* or *Xylella*, bacterial genera involved in cycling nitrogen as *Opitutus* and *Rhodanobacter*, and archaeal halophiles–*Haloferax* and archaeal mesophiles belonging to the phylum of *Thaumarchaeota*–*Nitrososphaera*. The appearance of those taxa did not seem to arise from simple PCR bias during generation of 16S rRNA gene amplicons, since used primers have a 4-fold lower chance to cover these taxa compared to the coverage score of all detected taxa according to PrimerProspector [53] (Table O in S1 File).

### Archaea and spore forming bacteria increase in terms of abundance and diversity in the microbiome of intact cells in the controlled cleanroom

As PCR bias was not an obvious explanation for this observation, the dataset was screened for trends with potential biological sources. This screening revealed a major increase of genera, which are able to form spores–*Ammoniphilus*, *Bacillus*, *Brevibacillus*, *Clostridium*, *Cohnella*, *Desulfosporosinus*, *Geobacillus*, *Paenibacillus*, *Planifilum*, *Sporosarcina*, *Terribacillus*, *Thermoactinomyces*, *Virgibacillus* and genera of the archaeal domain of life–*Haloferax* and Candidatus *Nitrososphaera*. Some of these detected genera with sequences from intact cells e.g. *Sporosarcina* or *Haloferax* were exclusively present in samples obtained from the cleanroom. The presence of these intact microbes in the microbiome of the cleanroom environment suggests that they were transferred from the gowning area into the cleanroom or even adapted themselves to these harsh environmental conditions.

Our observation that *Archaea* (*Haloferax* and Candidatus *Nitrososphaera*) occur as intact cells in a cleanroom setting, has not been previously reported. They may play an important role in microbiomes of the clean built environment, since most bacteria are either dead or have to outlast as dormant spores. *Archaea* of the phylum *Thaumarchaeota* were recently identified as an obvious contamination source in cleanrooms due to their close association to human skin [19]. Further on, the presence of intact halophiles of the archaeal domain of life in the cleanroom microbiome might arose from another human source–such as the human gut as reported by [54]. In addition we speculate that PMA introduced a shift into the amplicon population and was responsible for the increase of spore forming bacteria as this chemical cannot enter spores without treatments to increase permeability [31,55]. This shift is auxiliary for research focusing on the rare biosphere of extreme built environments and planetary protection in general (S6–S9 Figs and Table P and Q in S1 File), since sequences and taxa of great interest which may be problematic are relatively increasing not only in the aspect of abundance (archaea: 177 fold CCR, 2 fold UAF; potential spore formers: 219 fold CCR, 10 fold UAF; P = 4*10^−4^), but also in their respective diversity (archaea: 9 fold CCR, 3 fold UAF; potential spore formers: 3 fold CCR and UAF). Spore forming bacteria comprise most of those species which are suspected to be capable of surviving space travel and increase the risk of forward contaminations of other celestial bodies in the solar system [56]. Spore formers in general endanger food and pharmaceutical packaging in cleanrooms and pathogenic spore formers like *Bacillus anthracis* are a major threat if formulated as a bioweapon for harmful criminal interests or in the case of multi-resistant spore formers in hospitals.

### Functional redundancy of PICRUSt predicted functions

The possible functional properties of taxa detected in this marker gene analysis were predicted with PICRUSt. This analysis showed a decreased dissimilarity of samples irrespective of a sample grouping per category (controlled, uncontrolled, total, viable and all, see S10 Fig and Table R in S1 File). This observation suggests that despite different microbial profiles of the CCR and UAF environment, the functional capabilities of sampled communities might be more similar than previously expected, which would lead further to a strong functional redundancy of these indoor ecosystems.

### Closing remarks

Whereas microbial abundance could be reduced by strict maintenance procedures in the CCR (controlled cleanroom), microbial diversity remained almost constant compared to the UAF (uncontrolled adjoining facility, see Table 1 for a summary). In addition, the application of a PMA treatment to target the microbiome of intact cells and special groups like archaea or spore forming bacteria is very promising for other microbiome studies, since the loss of exclusive (only present with or without PMA treatment in CCR or UAF) abundance and diversity is very low.

**Table 1:** Summary of the microbial abundance and diversity detected in the spacecraft assembly cleanroom (controlled cleanroom–CCR) at NASA Jet Propulsion Laboratory, Pasadena, CA, USA and its surrounding uncontrolled adjoining facility—UAF. (Numbers for quantitative measures are given per m^2^).

analysis	CCR	UAF
Microbial abundance		
Microbial abundance total ATP	2.9*10^3^	7.5*10^5^
Microbial abundance total DNA (16S rrnDB)	4.4*10^5^	1.6*10^6^
Viable microbial abundance		
Viable microbial abundance intracellular ATP	1.4*10^3^	6.2*10^4^
Viable microbial abundance PMA treated DNA	6.2*10^3^	7.3*10^4^
Microbial diversity (at 10,011 sequences)		
Shannon-Wiener index (H’)	6.6	6.8
Phylogenic diversity (PD)	107.9	109.0
Species richness (chao 1)	5572.2	4607.1
Observed species	1673.9	1496.6
Coverage [%]	30.3	35.1
Viable microbial diversity (at 10,011 sequences)		
Shannon-Wiener index (H’)	5.8	6.0
Phylogenic diversity (PD)	37.0	48.7
Species richness (chao 1)	1147.4	1635.3
Observed species	472.1	690.4
Coverage [%]	46.5	44.3
Exclusive diversity		
Exclusive microbial diversity	20 genera	138 genera
Exclusive viable microbial diversity (+ PMA)	33 genera	38 genera
Shared OTUs		
Shared core OTUs	8.2%	49.6%
Shared viable core OTUs	52.4%	69.4%
Increase in terms of abundance and diversity		
Significant increase after PMA treatment	*Propionibacterium*, *Rickettsiales*, *Staphylococcus*, *Halomonas*	*Bacilli*, *Bacillales*, *Bacillus Shewanella*, *Nocardioidaceae*, *Staphylococcus*, *Halomonas*
Abundance increase of potential spore formers by PMA treatment	219 fold	10 fold
Abundance increase of Archaea by PMA treatment	177 fold	2 fold
Diversity increase of potential spore formers by PMA treatment	3 fold	3 fold
Diversity increase of Archaea by PMA treatment	9 fold	3 fold
Exclusive taxa		
Exclusive viable genera (diversity after PMA treatment)	11	11
Exclusive genera	21	121

Finally our approach also helped to identify sequences of not-yet described species from prior cleanroom studies (122 new detected genera with Illumina MiSeq compared to the following references based on 454 pyrosequencing, PhyloChip, traditional cloning and cultivation [23–26,30,57–60]. Reported genera should be considered for other clean built environments in the future, because they may serve as valuable indicator species for applications in the monitoring and validating levels of cleanliness in cleanrooms, operation theaters and hospital microbiomes.

## Conclusion

Our room maintenance e.g. strict control of rooms, cleaning and air filtration was shown to have a large impact on microbes in the built environment. Microbial abundance can be reduced to a large extent and most microbes simply could not withstand harsh conditions and did not proliferate as indicated by low levels of intracellular ATP and DNA from intact cells. However, the picture of microbial diversity is more complex: the diversity of microbes is only slightly reduced, since sequences of survival specialists like bacterial spore formers and archaeal halophiles and mesophiles appear to be enriched, as they are able to withstand harsh environmental conditions in the cleanroom. The bacteria detected are able to survive cleaning procedures and a low water as well as nutrient content by means of spore formation and waiting for favorable conditions. Similarly, human associated *Archaea* might be adapted to cleanroom conditions, since this domain of life is well known for their survival under many different extreme environmental conditions. Both groups of microbes have an obvious potential to interact with our human microbiome and affect our health either positively or negatively, since they are human associated and less controlled or stabilized by other species due to low amounts of intact microbes. Examinations of the ecological relevance of detected species and resolving their functions in the future should therefore aim to estimate the potential up–and downsides of strict microbial control, cleaning and reduction of microbial abundance and diversity for human health in the built environment.

## Supporting Information

S1 Fig Beta-diversity (weighted). (A) NMDS plot based on weighted unifrac distance matrix of rarefied OTUs to 10,011 sequences. Samples treated with PMA prior to DNA extraction are indicated by a plus symbol. CCR: controlled cleanroom. UAF: uncontrolled adjoining facility. Variances are explained per each axis (NMDS1 and NMDS2, Stress = 0.08). (B) HCAN based on average neighbor of weighted unifrac distances. Samples treated with PMA prior to DNA extraction are indicated by a plus symbol. CCR: controlled cleanroom. UAF: uncontrolled adjoining facility.
(TIF)

S2 Fig PCoA plot. PCoA plot based on Bray-Curtis distance matrix. Controls of each BiSKit sampler are shown as empty grey circles. CCR: controlled cleanroom samples are shown in red and pink (+ PMA treatment), labeled A, B, C. Samples from UAF: uncontrolled adjoining facility are shown in dark and light blue (+ PMA treatment), labeled 1 to 4. Variances are explained per each axis (PC1 and PC2).
(TIF)

S3 Fig Venn diagram. Venn diagram of detected genera in cleanroom (CCR) and gowning area (UAF). Treatment with PMA prior to DNA extraction is indicated by a plus symbol (+). Numbers indicate amount of detected genera.
(TIF)

S4 Fig OTU heatmap. Heatmap based on taxa, which are part of the core microbiome of all samples. Color code from blue via white to red (0–50–100%) gives relative amount [%] of respective taxonomic group. Table was sorted according to resulting P-values (p) of an ANOVA test (significant (p) at an alpha of 0.05 are highlighted in bold). (p) were corrected with false discovery rate (fdr (p)) and bonferroni (bonf. (p)). Table was rarefied to 2329 OTUs. Samples treated with PMA prior to DNA extraction are indicated by a plus symbol. CCR—controlled cleanroom. UAF:- uncontrolled adjoining facility.
(TIF)

S5 Fig Bar chart. Chart demonstrates high amount of rare abundant OTUs. 99% of the diversity is expressed in only 1% of the total number of reads. Bars in the chart are resolved to CCR (controlled cleanroom) and UAF (uncontrolled adjoining facility) respectively.
(TIF)

S6 Fig Relative proportion of OTUs resolved to genus level. Relative proportion of OTUs grouped to potential spore forming bacteria and archaea, in presence and absence of prior PMA treatment. Panel A shows increase of OTUs assigned as spore forming bacteria after PMA treatment of samples. Panel B shows increase of OTUs assigned to archaea after PMA treatment of samples (panel B shows only one percent of the whole y-axis). Archaea P-value = 0.522; other bacteria P-value = 0.977; potential spore forming bacteria P-value = 0.000448 (ANOVA, alpha = 0.05). PMA treatment of samples is indicated by a plus symbol. CCR–controlled cleanroom, UAF–uncontrolled adjoining facility.
(TIF)

S7 Fig Relative proportion of exclusive OTUs. Exclusive OTUs (either only present after or prior to PMA treatment) resolved to genus level and grouped to potential spore forming bacteria and archaea. Panel A shows increase of exclusive OTUs assigned as spore forming bacteria after PMA treatment of samples. Hence, PMA treatment of a sample results in more OTUs for spore formers towards a negligible quantity of OTUs getting lost by PMA treatment. Panel B shows increase of OTUs assigned to archaea after PMA treatment of samples in CCR (panel B shows only half of the whole y-axis). Archaea P-value = 0.311; other bacteria P-value = 1.15*10^−6^; potential spore forming bacteria P-value = 0.148 (ANOVA, alpha = 0.05). PMA treatment of samples is indicated by a plus symbol. CCR–controlled cleanroom, UAF–uncontrolled adjoining facility.
(TIF)

S8 Fig Relative proportion of microbial diversity. Microbial diversity (taxa) resolved to genus level and grouped to potential spore forming bacteria and archaea, in presence and absence of prior PMA treatment. Panel A shows increase of OTUs assigned as spore forming bacteria and archaea after PMA treatment of samples. Panel B shows magnification of panel A (panel B shows only one fourth of the whole y-axis). PMA treatment of samples is indicated by a plus symbol. CCR–controlled cleanroom, UAF–uncontrolled adjoining facility.
(TIF)

S9 Fig Relative proportion of exclusive microbial diversity. Exclusive microbial diversity (taxa—either only present after or prior to PMA treatment) resolved to genus level and grouped to potential spore forming bacteria and archaea. Panel A shows increase of exclusive diversity assigned as spore forming bacteria and archaea after PMA treatment of samples. Hence, PMA treatment of a sample results in higher diversity for spore formers towards a negligible quantity of diversity getting lost by PMA treatment. Panel B shows magnification of panel A (panel B shows only one fourth of the whole y-axis). PMA treatment of samples is indicated by a plus symbol. CCR–controlled cleanroom, UAF–uncontrolled adjoining facility.
(TIF)

S10 Fig 3D—PCoA plot. PCoA plot based on Bray-Curtis distance matrix of PICRUSt predicted functions from marker gene analysis. CCR: controlled cleanroom samples are shown in red and pink (+ PMA treatment). Samples from UAF: uncontrolled adjoining facility are shown in dark and light blue (+ PMA treatment). Variances are explained per each axis (PC1, PC2, and PC3).
(TIF)

S1 File Supplementary tables. Table A: Handheld ATP counts to reveal the gross contamination of the surface to be sampled. Table B: Statistics on raw and quality filtered reads. Table C: Summary of ANOVA results on all tested categories. Table D: Summary of ANOVA results on category "ATP" with detailed results of the Tukey test. Table E: Summary of ANOVA results on category "qPCR" with detailed results of the Tukey test. Table F: Summary of ANOVA results on categories "ATP" and "qPCR" with detailed results of the Tukey test. Table G: Summary of ANOVA results on categories "ATP" and "PMA". Table H: Summary of ANOVA results on categories "total" and "living". Table I: Microbial diversity in respect of type of the data set. Table J: OTU table of CCR and UAF samples resolved to genus level. Table K: Detected only by the use of PMA in CCR and UAF. Table L: Analysis of variances on taxa displayed in the heatmap of the core microbiome (see Fig 5). P-values were corrected for false discovery rate (fdr) and bonferroni corrected. Table M: Tukey test on ANOVA results shown in Table L. Significant P-values are highlighted in bold. Table N: Taxonomic resolution of single and doubletons till genus and family level. Table O: Coverage of primers 515f and 806r (used for Illumina MiSeq 16S rRNA gene Amplicons). Table P: Analysis of variances (ANOVA) results with Tukey test for different groups of genera (archaeal genera, potential spore forming genera and other genera). Table Q: Analysis of variances (ANOVA) results with Tukey test for different groups of exclusive (either present with or without PMA treatment in CCR or UAF) genera (archaeal genera, potential spore forming genera and other genera). Table R: Relative proportions of PICRUSt pridicted functions with its nearest sequence taxonomic index (weighted NSTI) of 16S rRNA marker gene analysis of CCR and UAF. Samples were grouped into main categories: controlled (CCR samples), uncontrolled (UAF samples), total (all untreated samples), viable (all PMA treated samples) and all (all samples).
(XLSX)
