# Supplementary figures and images for: Cleanroom Maintenance Significantly Reduces Abundance but Not Diversity of Indoor Microbiomes
Source: PLoS One. 2015 Aug 14;10(8):e0134848. doi: 10.1371/journal.pone.0134848 (PMC4537314; doi:10.1371/journal.pone.0134848)

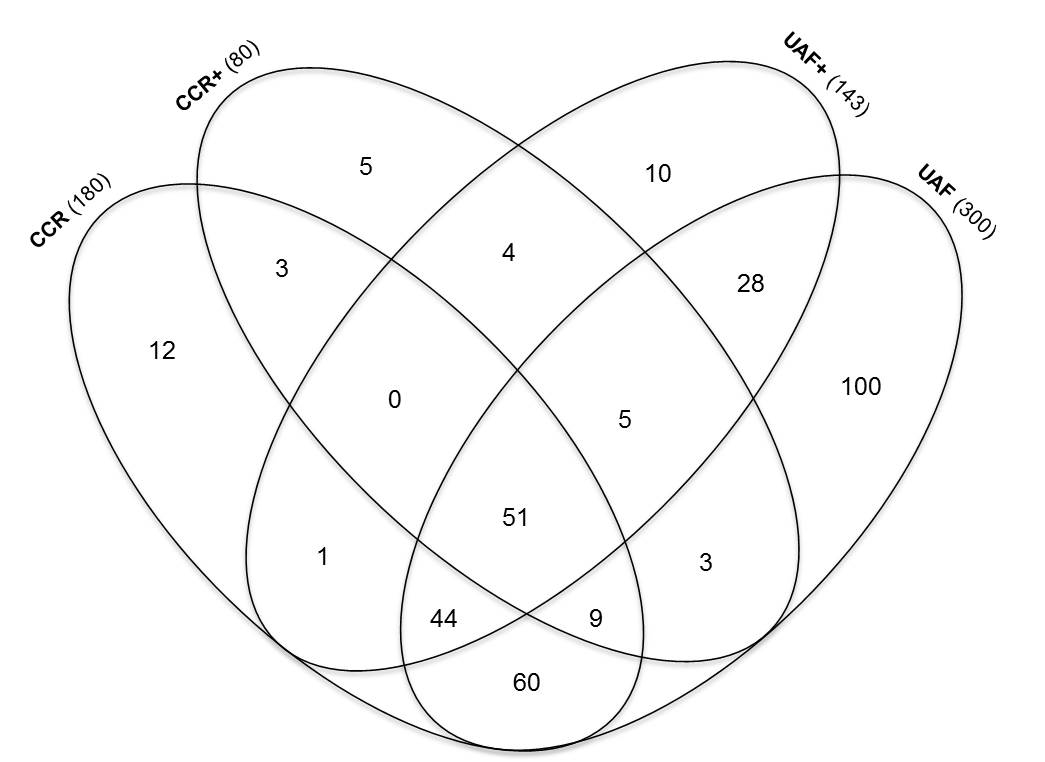

Supplement: S3 Fig — Venn diagram of detected genera in cleanroom (CCR) and gowning area (UAF). Treatment with PMA prior to DNA extraction is indicated by a plus symbol (+). Numbers indicate amount of detected genera. (TIF) [file pone.0134848.s003.tif]

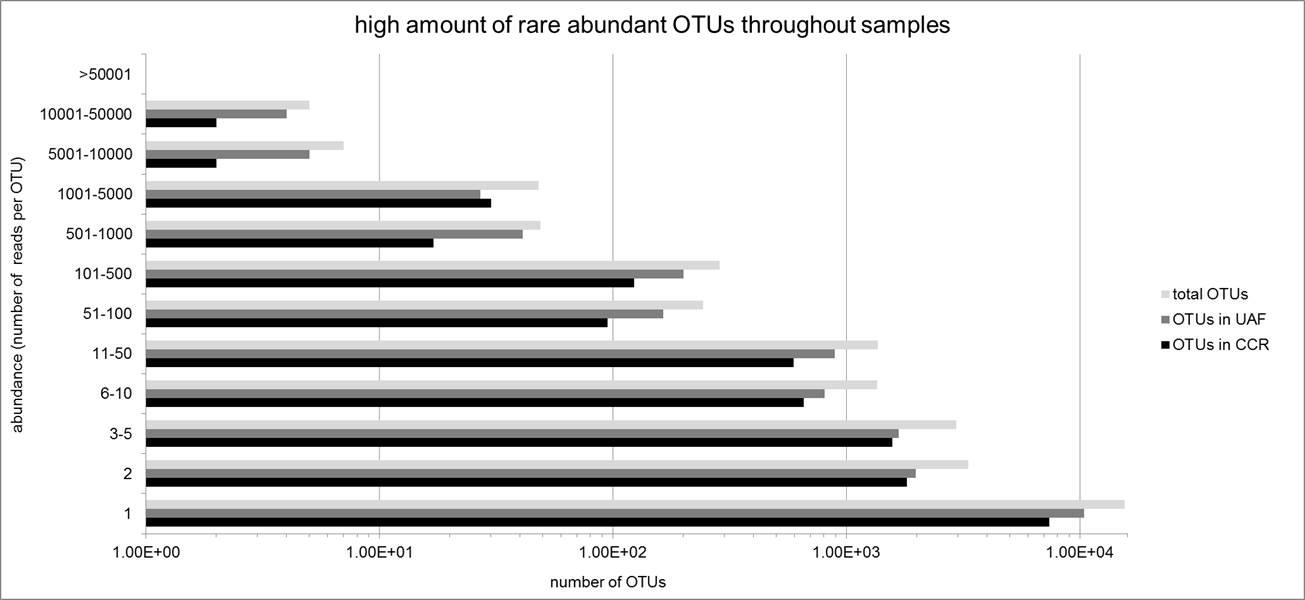

Supplement: S5 Fig — Chart demonstrates high amount of rare abundant OTUs. 99% of the diversity is expressed in only 1% of the total number of reads. Bars in the chart are resolved to CCR (controlled cleanroom) and UAF (uncontrolled adjoining facility) respectively. (TIF) [file pone.0134848.s005.tif]

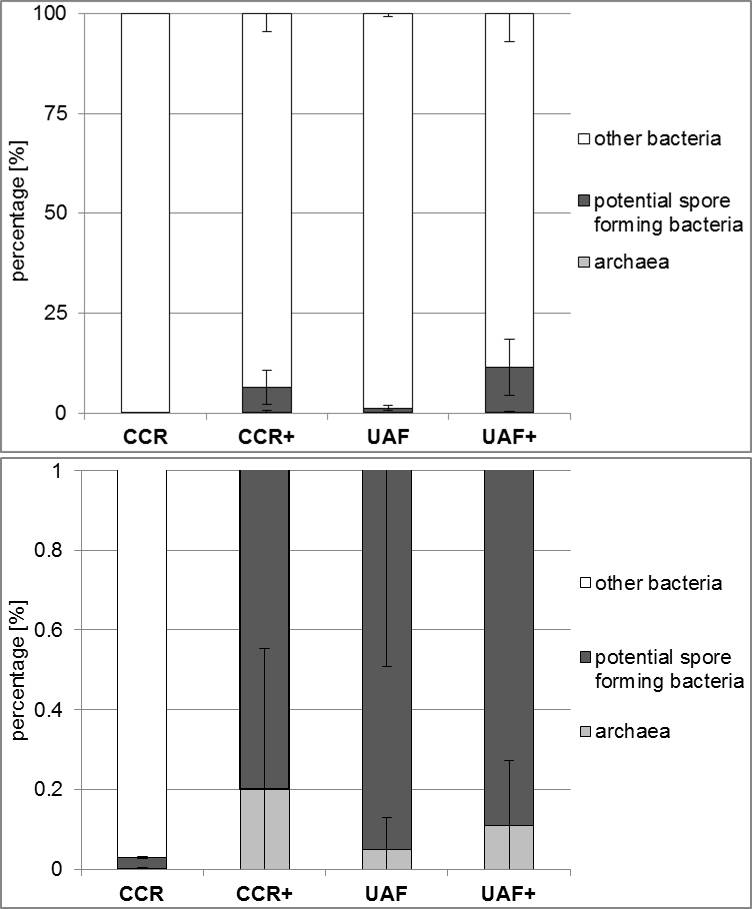

Supplement: S6 Fig — Relative proportion of OTUs grouped to potential spore forming bacteria and archaea, in presence and absence of prior PMA treatment. Panel A shows increase of OTUs assigned as spore forming bacteria after PMA treatment of samples. Panel B shows increase of OTUs assigned to archaea after PMA treatment of samples (panel B shows only one percent of the whole y-axis). Archaea P-value = 0.522; other bacteria P-value = 0.977; potential spore forming bacteria P-value = 0.000448 (ANOVA, alpha = 0.05). PMA treatment of samples is indicated by a plus symbol. CCR–controlled cleanroom, UAF–uncontrolled adjoining facility. (TIF) [file pone.0134848.s006.tif]

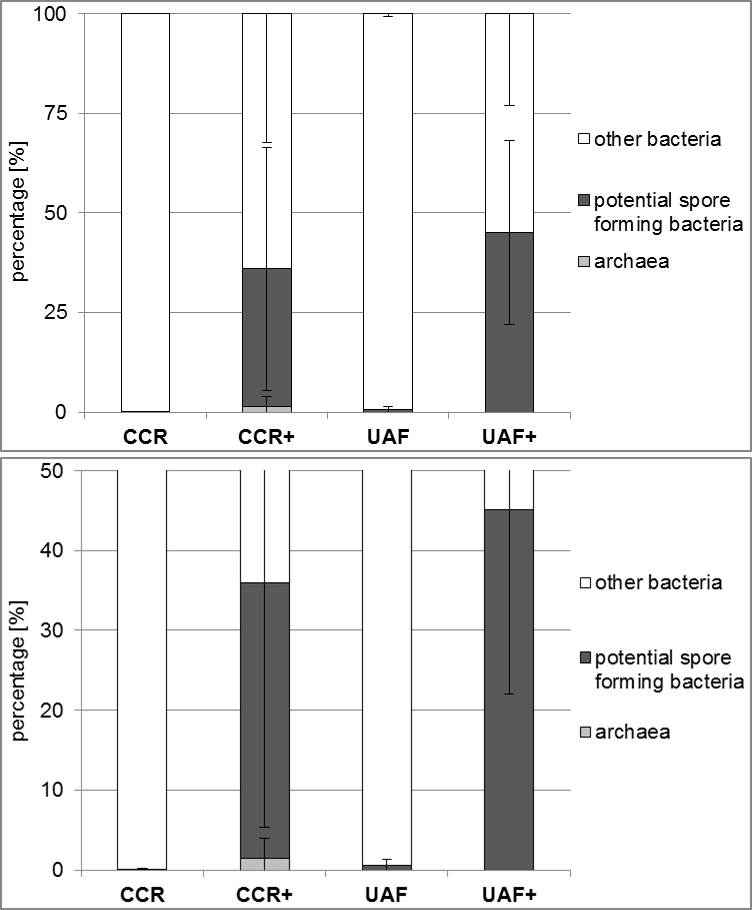

Supplement: S7 Fig — Exclusive OTUs (either only present after or prior to PMA treatment) resolved to genus level and grouped to potential spore forming bacteria and archaea. Panel A shows increase of exclusive OTUs assigned as spore forming bacteria after PMA treatment of samples. Hence, PMA treatment of a sample results in more OTUs for spore formers towards a negligible quantity of OTUs getting lost by PMA treatment. Panel B shows increase of OTUs assigned to archaea after PMA treatment of samples in CCR (panel B shows only half of the whole y-axis). Archaea P-value = 0.311; other bacteria P-value = 1.15*10−6; potential spore forming bacteria P-value = 0.148 (ANOVA, alpha = 0.05). PMA treatment of samples is indicated by a plus symbol. CCR–controlled cleanroom, UAF–uncontrolled adjoining facility. (TIF) [file pone.0134848.s007.tif]

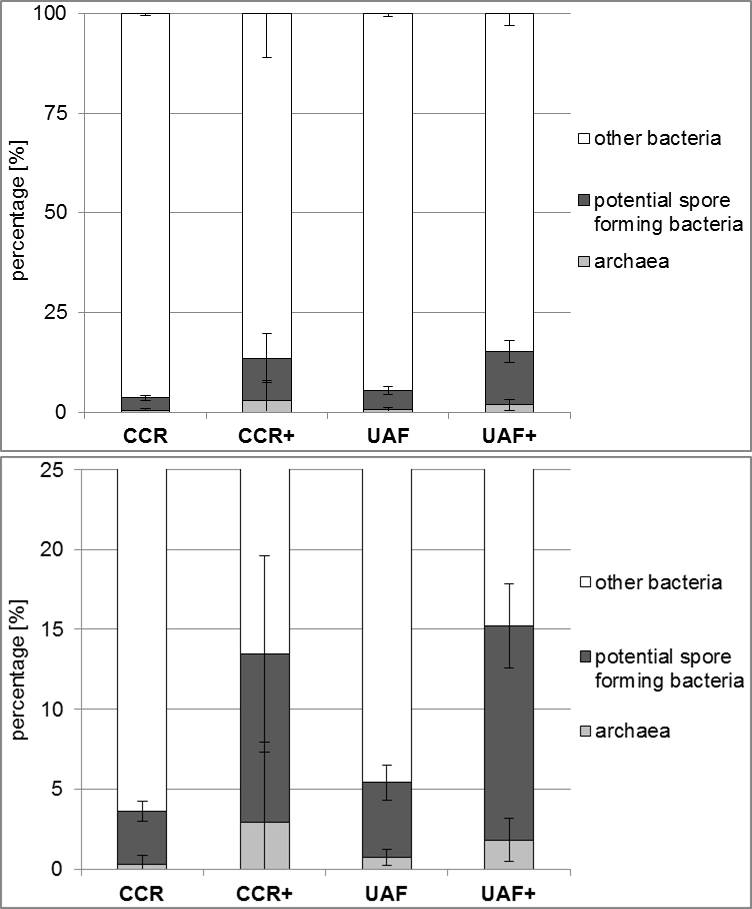

Supplement: S8 Fig — Microbial diversity (taxa) resolved to genus level and grouped to potential spore forming bacteria and archaea, in presence and absence of prior PMA treatment. Panel A shows increase of OTUs assigned as spore forming bacteria and archaea after PMA treatment of samples. Panel B shows magnification of panel A (panel B shows only one fourth of the whole y-axis). PMA treatment of samples is indicated by a plus symbol. CCR–controlled cleanroom, UAF–uncontrolled adjoining facility. (TIF) [file pone.0134848.s008.tif]

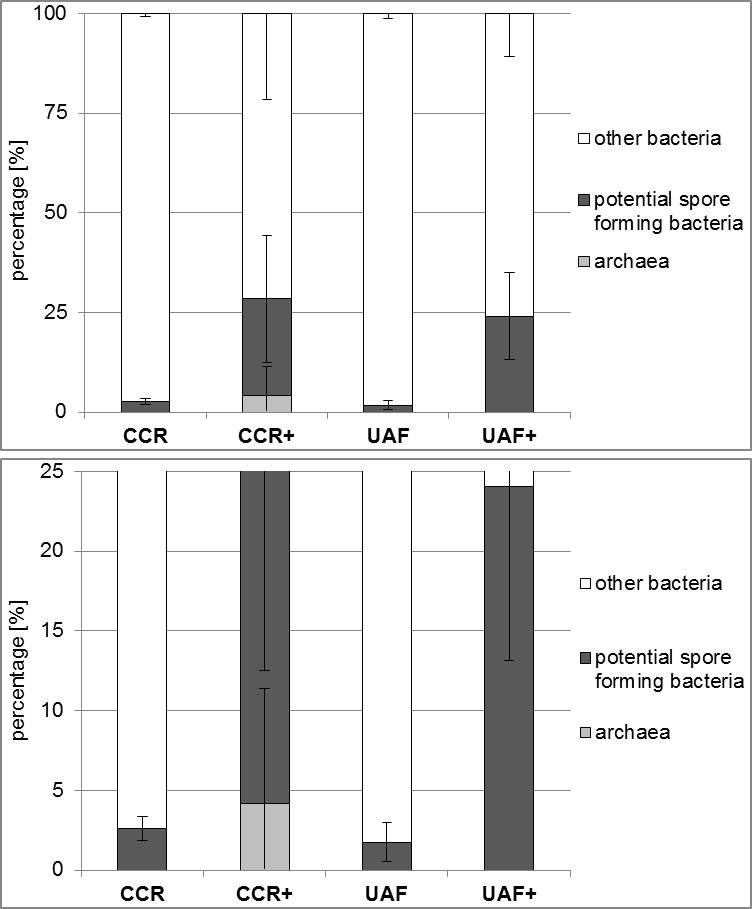

Supplement: S9 Fig — Exclusive microbial diversity (taxa—either only present after or prior to PMA treatment) resolved to genus level and grouped to potential spore forming bacteria and archaea. Panel A shows increase of exclusive diversity assigned as spore forming bacteria and archaea after PMA treatment of samples. Hence, PMA treatment of a sample results in higher diversity for spore formers towards a negligible quantity of diversity getting lost by PMA treatment. Panel B shows magnification of panel A (panel B shows only one fourth of the whole y-axis). PMA treatment of samples is indicated by a plus symbol. CCR–controlled cleanroom, UAF–uncontrolled adjoining facility. (TIF) [file pone.0134848.s009.tif]

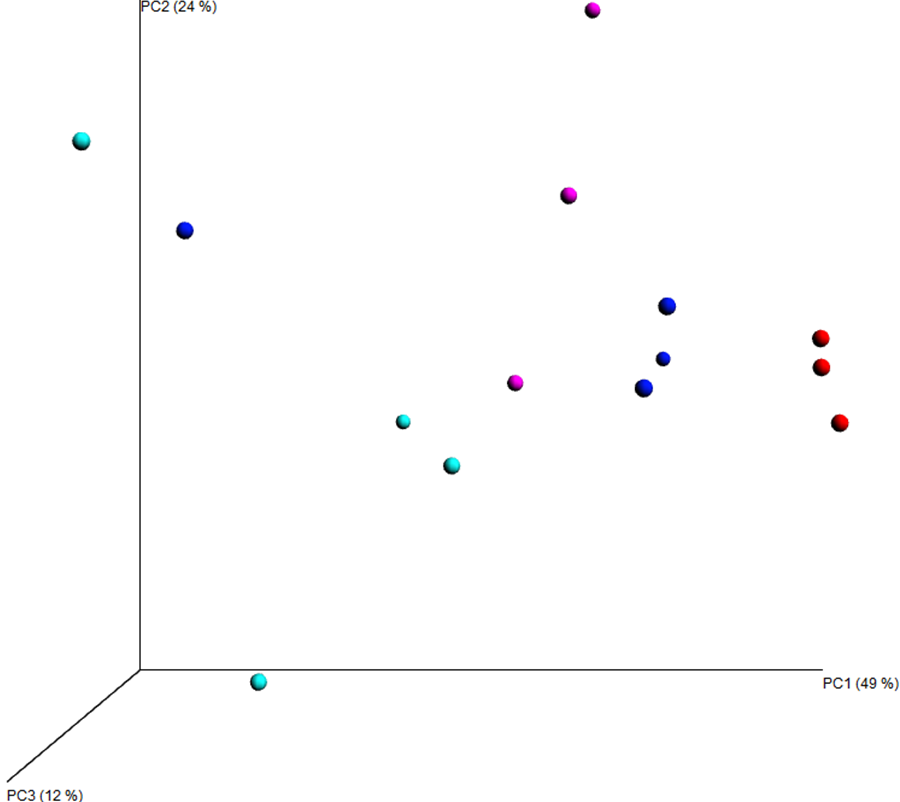

Supplement: S10 Fig — PCoA plot based on Bray-Curtis distance matrix of PICRUSt predicted functions from marker gene analysis. CCR: controlled cleanroom samples are shown in red and pink (+ PMA treatment). Samples from UAF: uncontrolled adjoining facility are shown in dark and light blue (+ PMA treatment). Variances are explained per each axis (PC1, PC2, and PC3). (TIF) [file pone.0134848.s010.tif]
